# Embryology and genetics of primary vesico-ureteric reflux and associated renal dysplasia

**DOI:** 10.1007/s00467-006-0390-1

**Published:** 2007-06-01

**Authors:** Luisa Murer, Elisa Benetti, Lina Artifoni

**Affiliations:** 1 Paediatric Nephrology, Dialysis and Transplant Unit, Department of Paediatrics, University of Padova, Padova, Italy; 2 Laboratory of Paediatric Nephrology, Department of Paediatrics, University of Padova, Padova, Italy; 3 Paediatrics, University of Padova, Via Giustiniani 3, Padova, 35128 Italy

**Keywords:** Vesico-ureteric reflux, Embryology, Genetics, Kidney development

## Abstract

Congenital anomalies of the kidney and urinary tract, as well as primary vesico-ureteric reflux (VUR) and associated renal dysplasia, are the most relevant causes of end-stage renal failure in the pediatric population. In vivo and in vitro experimental studies have allowed the identification of several genes involved both in ureteric bud branching, ureteric elongation and insertion into the bladder, and in nephrogenesis. It has been proposed that both renal and ureteral abnormalities, as well as the associated renal hypo-dysplasia, may derive from a common mechanism as the result of a dysregulation of the normal developmental program. The large homologies between mice and the human genome suggest that the same genes could be involved both in rodent and human VUR. Furthermore, epidemiological observations suggest that not only syndromic but also isolated VUR is an inherited trait. Linkage analysis for homologous mouse genes in humans, genome-wide linkage studies in multigenerational families and association studies by polymorphisms support the hypothesis that VUR is genetically heterogeneous and is caused by a number of different genes acting with random environmental effects. The present teaching paper is an overview of the embryology and genetics of primary VUR and associated congenital reflux nephropathy.

## Introduction

**Fig. 1:**
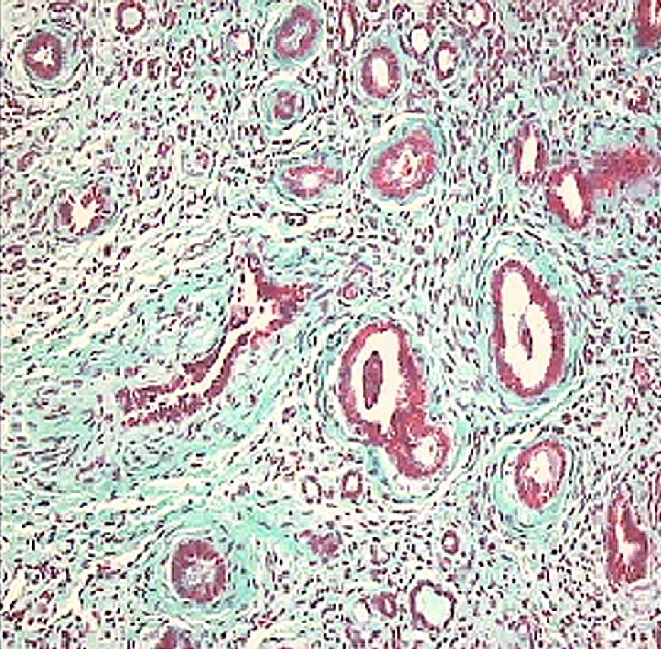
Vesico-ureteric reflux-associated renal dysplasia. Dysplastic kidneys are characterized by the presence of renal parenchymal areas with various degrees of differentiation together with areas of undifferentiated nephrogenic mesenchyme and cartilaginous metaplasia, with or without different localized and extended tubular, ductal or glomerular cysts (Masson’s Trichromic dye, ×40)

Primary vesico-ureteric reflux (VUR) is a congenital defect of the urinary tract that causes urine to flow retrogradely from the bladder to the kidneys and it is not associated with any underlying neuromuscular or obstructive phenomenon [1]. Galen and Leonardo Da Vinci firstly described the oblique entry of ureter into the bladder that prevents the back-up of urine during bladder voiding. Later animal studies and human observations showed that developmental defects of the vesico-ureteric junction determining VUR include inadequate length of the intravesical ureter; the defective muscle layer of the trigone that encompasses the ureter adjoining the bladder and ectopic ureteral orifices within the trigone. VUR is a risk factor for upper urinary tract infections (UTI), causing pyelonephritis and renal scarring. Recurrent scarring and subsequent chronic renal damage may lead to progressive renal failure. VUR prevalence in the pediatric population is 1%. NAPRTCS and European registries report that 8% of patients affected by VUR develop end-stage renal failure as a result of “reflux nephropathy”, which accounts for 25% of end-stage renal disease [1–3]. Probably, many other individuals with VUR hidden in the population are affected by renal dysplasia and/or hypoplasia, which represent the major cause of end-stage renal failure. However, the prevention of UTI by clinical management (antibiotic prophylaxis) and/or by surgical correction of VUR have not significantly reduced the incidence of renal failure in these patients. Non-functioning renal units associated with VUR and end-stage renal failure are mostly hypo-dysplastic kidneys. The histological picture of dysplastic kidneys is characterized by the presence of renal parenchymal areas with various degrees of differentiation together with areas of undifferentiated nephrogenic mesenchyme and cartilaginous metaplasia, with or without different localized and extended tubular, ductal or glomerular cysts (Fig. 1). It is still controversial whether renal dysplasia is due to poor nephrogenic differentiation resulting from the back pressure effect of urine reflux on the developing fetal kidney. The degree of renal dysplasia does not correlate well with the severity of reflux. More recently, it has been proposed that both renal and ureteral abnormalities may derive from a common mechanism as the result of a dysregulation of the normal developmental program occurring very early during embryogenesis. Nephrourogenetic programming requires a complex sequential and temporal regulation of genes in terms of activation and repression. Experimental gene knock-out models have enabled the identification of some genes associated with VUR and reflux nephropathy, and mutations of murine-homologous human genes are responsible for syndromic forms of VUR-associated hypo-dysplasia.

## Embryology of the kidney and urinary tract: the developmental theory of vesico-ureteric reflux

**Fig. 2:**
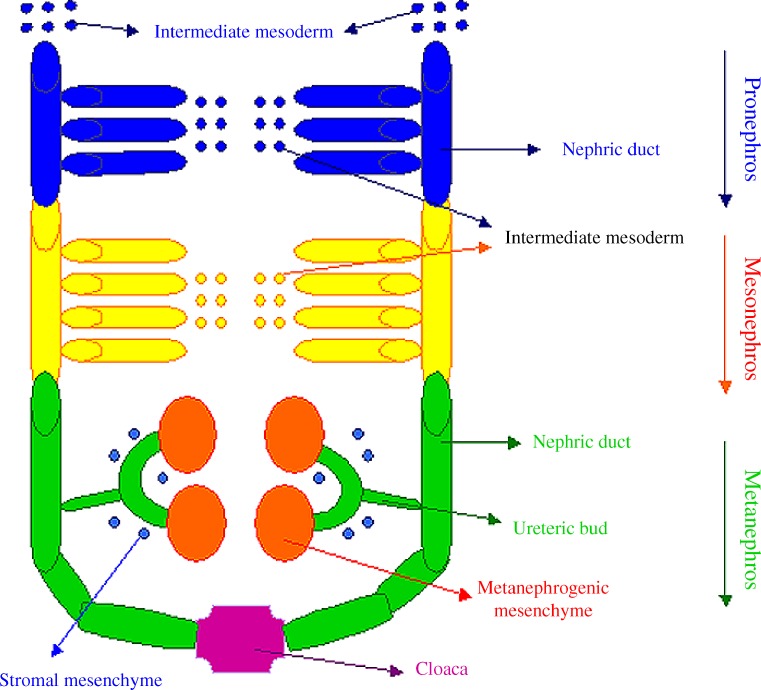
Schematic representation of early events in kidney development (modified from [30])

In humans, as in other mammalian species, the kidney and ureter derive from a mesodermic tissue, the metanephros, which develops in close proximity to the mesonephric Wolffian duct, which extends to fuse with the cloaca, the urinary bladder precursor, and drains the transitory kidneys (pro- and mesonephroi; Fig. 2). The metanephros consists of two components: the ureteric bud and the metanephric mesenchyme. The former gives rise to the collecting ducts, the renal pelvis, the ureter, and the bladder trigone, while the latter differentiates into tubules (from distal tubules up to the glomerulus) and renal stroma. The development of both the components depends on a series of reciprocal inductive signals: at the end of the 4th week of gestation, metanephric mesenchyme induces the nearby Wolffian duct to evaginate an epithelial tube, the ureteric bud. This invades the metanephric mesenchyme, which in response condenses, proliferates, and undergoes an epithelial transformation at approximately the 5th week of gestation. In turn, the distal end of the ureteric bud branches to form the collecting system in response to contact with the stimulated metanephric mesenchyme. The proximal end of the ureteric bud remains attached to the mesonephric duct and elongates, becoming the distal ureter. The common nephric duct, the segment of the mesonephric duct that lies between the ureter and urogenital sinus or primitive bladder, undergoes programmed cell death. This permits the ureter to begin its incorporation into the bladder, during which it acquires a tunnel and a muscle layer that connects to the trigone [2].

In children with ureteral malformations, Mackie and Stephens observed two kinds of morphologic correlation: between the location of the ureteral orifice and the abnormalities of the ureter (i.e., VUR); and between the location of the ureteral orifice and the degree of kidney dys- or hypoplasia (cited in [4]).

**Fig. 3:**
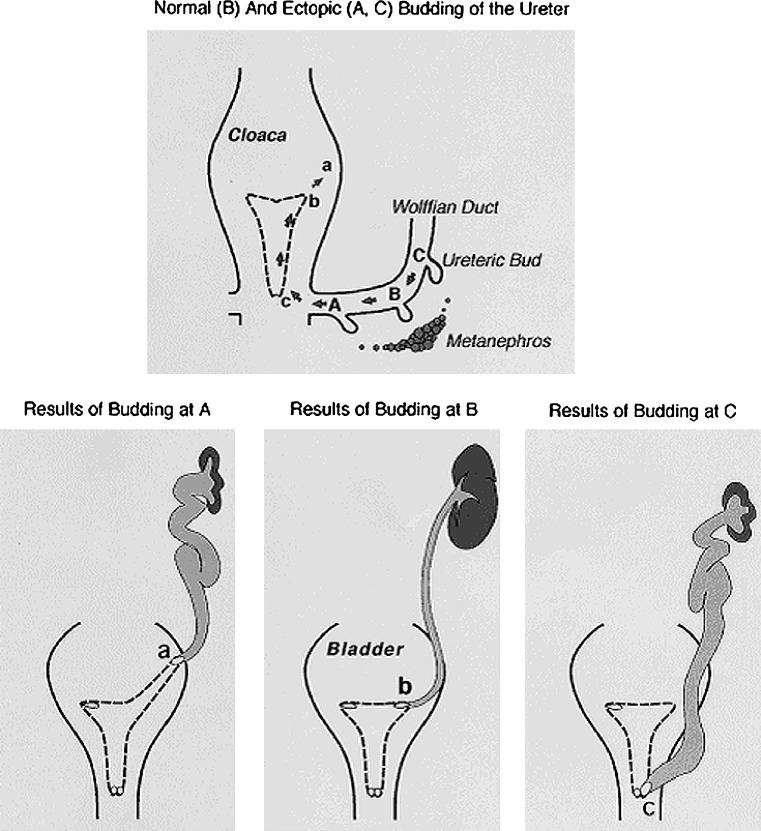
Dynamics of the kidney and ureteral development according to the “bud theory”. Relationship between ureteric bud position on the Wolffian duct to the nephrogenic blastema and orifice positions in the bladder and urethra. *A* Ectopic bud orifice on the lateral and cranial end of the extended hemitrigone (*site a*), resulting in VUR. *B* Normal ureteric bud orifice *B* on the corner (*site b*). *C* Ectopic bud orifice on the caudal extremity of the bladder. The metanephric mesenchyme is well differentiated when interacting with a bud at the normal site *B* but sparse and poorly differentiated around bud *A* and *C* (from [4])

In the developing embryo, the ureteral orifice travels from its original budding site on the Wolffian duct to the final destination into the bladder as the Wolffian duct region is absorbed into the developing bladder. The final site of the ureteral orifice depends on the position where the ureteric bud exits from the mesonephric duct, which in turn depends on the time and location of the contact with the metanephric mesenchyme. For example, if the ureteric bud develops at a site too caudal along the Wolffian duct, its final location within the bladder will be abnormally cranial to the normal site (Fig. 3). This aberrancy results in the poor development of a “short” ureterovesical valve and VUR. Concurrently, the normal interaction between the ureteric bud and the metanephric blastema required for normal renal parenchyma growth and differentiation is also disrupted. The ureteral budding from an aberrant site contacts the metanephric mesenchyme at a point where mesenchymal cells are scarce, resulting in formation of hypoplastic and dysplastic kidneys. Renal hypo-dysplasia is not the result of urine reflux, but both kidney and ureter malformation derive from an aberrant contact between the ureteric bud and the metanephric blastema [4]. In the last decade, in vitro (organ culture) and in vivo (knock-out animal) studies and gene expression profile analysis on developing kidneys have provided essential data for the generation of hypotheses regarding the molecular control of nephrogenesis, enabling the identification of hundreds of implicated molecules, including growth/survival factors, cell adhesion molecules, and transcription factors.

## Molecular regulation of nephrogenesis and candidate genes of VUR

**Fig. 4:**
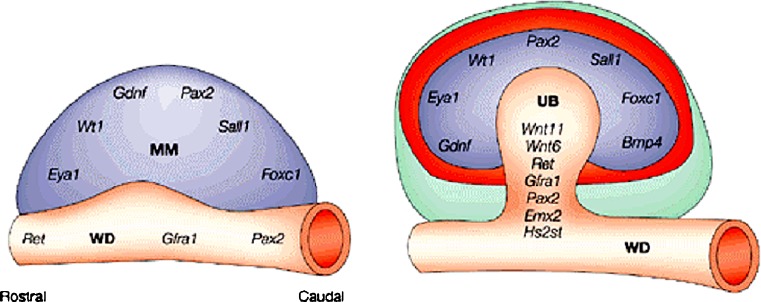
Overview of genes involved in ureteric bud induction. *MM* metanephric mesenchyme, *UB* ureteric bud, *WD* wolffian duct. See text for details (modified from [8])

*Primum movens* for metanephros development is the formation of the metanephrogenic mesenchyme [5]. This event depends on the prior activation in the intermediate mesoderm of transcription factors produced by normally formed pronephros and mesonephros, such as *Lim-1*, *Pax-2*, *Eya-1*, and *Foxc-1* (Fig. 4) [6]. Once it has formed, the mesenchyme secretes *Gdnf* (glial-derived neurotrophic factor), inducing the nearby Wolffian duct to give out the ureteric bud, which invades the metanephric blastema and begins its branching and elongation. In *lim-1*−/− mice, the intermediate mesenchyme is disorganized and fails to express other proteins necessary for kidney development such as *Pax-2*. This is a transcription factor expressed in mesonephros and later in metanephros. *Pax-2*−/− mice fail to form any kidney [7]. In knock-out mice for the transcriptional co-activator *Eya-1*, the caudal end of the intermediate mesenchyme never produces *Gdnf* and so never initiates metanephric development. *Foxc-1*−/− mice show a disorganization of mesonephroi and their metanephrogenic mesenchyme forms too anteriorly, causing the ureter to be positioned further forward than usual, or an additional ureter to form on the same side. More recently, it has been reported that mesenchymal expression of *Sall1* transcription factor is necessary for early inductive events in murine kidney [6]. When metanephric mesenchyme is formed, the primary signal that it emanates is the growth factor *Gdnf*, which stimulates the ureteric bud out-growing from the Wolffian duct through its receptor *c-Ret* (retinoic acid receptor), a tyrosine kinase, and its *Gfr-1α* coreceptor, both expressed by the ureteric bud. This signaling system continues to operate after bud induction, sustaining its intra-mesenchymal arborization. The importance of the *Gdnf/Ret* pathway has been confirmed by the absence of kidneys in homozygous null mutant mice and by the absence of ureteric out-growth in experiments with blocking antibodies to *Gdnf* in culture of whole metanephric rudiments. Conversely, the addition of *Gdnf* to cultured metanephroi induces ectopic ureters. The receptor tyrosine kinase antagonist *Spry1* negatively regulates the *Gdnf/Ret*-signaling pathway, such that in *Spry1*−/− mice the mesonephric duct is more sensitive to *Gdnf* and develops multiple ureteric buds [5, 6, 8]. In the *HoxB7/Ret* transgenic mouse *Ret* is over-expressed and the kidneys are small and cystic with grossly dilated ureters and a shorter intravesical tunnel, into which urine flows retrogradely from the bladder, resulting in postnatal VUR. Mice lacking either *Ret*, or an enzyme required for retinoic acid synthesis have malformed kidneys and defects in the distal ureter: in males, the ureter remains attached to the mesonephric duct, which forms the deferent duct in adults, while it inserts into the uterus in females. Signaling through vitamin A and *Gdnf/Ret* is required for the common nephric duct to contact the urogenital sinus and undergo apoptosis during the formation of the vesico-ureteric junction.

Expression of *Ret* by the ureteric bud requires other transcription factors such as *Emx-2*; bud development also depends on the mesenchyme-derived growth factor *Hgf* with its tyrosine kinase receptor *c-Met*. Members of the bone morphogenetic protein family exert varied effects on ureteric bud development: *Bmp-2* acts as a powerful inhibitor of ureteric bud branching, restricting ramification to the terminal part of the bud; *Bmp-4* expression in the stroma around the ureteric bud and along the stalk of the ureteric bud prevents the ureteric bud from forming too early, or anteriorly along the Wolffian duct and bifurcating before it invades the mesenchyme. Heterozygous *bmp-4*+/− mice are animal models of vesico-ureteric junction abnormalities, which are often associated with bifid ureters. Many other growth factors, such as *Tgf-β* and *Fgf*, and some components of the extracellular matrix, such as glycosaminoglycans and proteins, partly secreted by the bud and partly by the surrounding mesenchyme, have been found to participate in ureteric development.

**Fig. 5:**
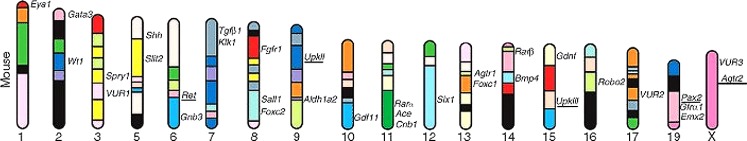
Candidate genes for vesico-ureteric reflux and urinary tract malformations and their genomic loci in mice (modified from [3])

Recently, 20 different transcripts have been identified as potentially important in metanephros development by microarray analyses on the rat tips of the ureteric tree. One of these is the cytokine-like factor 1 (*Cfl-1*), which induces mature nephron structures expressing glomerular and tubular markers when applied to isolated rat metanephric mesenchyme. The isolation of metanephrogenic mesenchyma from the ureteric bud would result in its death, but this fate is reversed by factors secreted by the bud, such as *Tgf-α*, *Timp-2*, *Egf*, and *Fgf-2*. Other factors, such as *Lif* and *Tgf-β2*, cooperate with the previously induced clumps of mesenchyme cells to differentiate into nephrons, while *Bmp-7* appears to lead them instead to form stroma [9]. As nephrons form, they express transcription factors such as *Pax-2* and *Hoxa-11*, and condense and secrete *Wnt-4*, which acts in an autocrine loop to stimulate its own synthesis and is required for mesenchymal cells to differentiate into epithelia. As well as acquiring their basic epithelial character, nephron cells have to differentiate to produce specialized functional regions. Maintained expression of *Pax-2* is necessary for nephron tubule elongation, but its inhibition by *Wt-1* is fundamental for normal glomerular development. While *Pax-2* homozygous null mutant mice develop renal hypo-dysplasia associated with various urinary tract malformations such as VUR, *Pax-2* over-expression induces hyper-proliferative tubular cysts and a failure of mature glomerular development [10]. Many other proteins have been recognized to be involved in region differentiation of the nephron, such as *Lmx-1b*, *Notch-2*, *Jagged-1*, and *Hnf-1*, but a larger number of molecules with similar activities are still to be identified [3]. Figure 5 summarizes candidate genes for VUR and urinary tract malformations, and their genomic loci in mice.

Briefly, experimental models suggest that:

1. Developmental anomalies of the urinary tract are due to alterations perturbing the complex gene network that controls nephrourogenesis

2. The different phenotypes of renal and ureteric malformations may depend on the stage of nephrogenesis at which the alteration of the developmental program occurs: renal agenesis should be the result of the complete lack of metanephric induction by the ureteric bud; different degrees of dysplasia associated with urinary tract anomalies, such as VUR, should originate from the disruption of the normal ureteral branching and elongation, and the epithelial differentiation processes

3. A mutation affecting a single gene may result in different phenotypes, and mutations of different genes can result in the same disease

4. Dysregulation of an upstream gene of a metabolic pathway may alter the function of one or more downstream genes of that very metabolic pathway or of other related ones, involved in the determination of a specific phenotype (modifier gene)

It has also been shown that not only a lack of transcription of a gene, but also its overexpression, may be responsible for a disease.

## Genetic basis of VUR

On the basis of human homolog gene knock-out mice models, which have identified a series of genes responsible for the normal or abnormal development of the urinary tract, a genetic basis for VUR and the associated renal dysplasia may be hypothesized in humans. VUR can occur as part of complex syndromes with an inherited pattern [11]. The genetic hypothesis is also supported by epidemiological evidence: isolated VUR has a high incidence in the population with ethnic differences between affected and non-affected individuals (reduced incidence in African Americans); monozygotic twins show 80–100% concordance for VUR compared with 35–50% concordance in dizygotic twins; 30–50% of the siblings of affected children have VUR and familial clustering of isolated VUR is well described.

In humans, as in mice, renal tract disorders occur with multiorgan malformation syndromes commonly affecting eyes and the central nervous, cardiovascular, and skeletal systems. Although individually rare, malformation syndromes collectively account for significant morbidity. Some are associated with gross chromosomal anomalies such as monosomy of chromosome X, trisomy of chromosomes 13, 18, and 21; deletion of chromosomes 4p, 5p, and 11p; and several microdeletions such as chromosome 7q, 22q11, and 17p11.2. However, cytogenetic rearrangements are absent in most children born with syndromic urinary tract malformations. Table 1 summarizes human syndromes with VUR with their own inheritance pattern. Several syndromes have an identified gene (mice homolog), whose protein and function are also well known.

**Table 1:** Human syndromes with vesico-ureteric reflux (*VUR*)

Syndromes	Kidney and urinary tract phenotype	Other associated abnormalities	Inheritance pattern	Disrupted gene product
Acrorenal (Siegler)	VUR, renal ectopia, HN, ureteral atresia/stenosis	SS, hypoplastic radii/ulnae/humeri, oligodactyly	U	
Adrenal hypoplasia-MR	VUR, HN, ureteral atresia/stenosis	Aminoaciduria, MR, muscular dystrophy, visual abnormality	XL	
Bardet-Biedl	VUR, renal cysts/dysplasia, duplicity, HN, nephritis/sclerosis	Obesity, polysyndactyly, MR, retinopathy, hypogonadism	AR	*BBS 1-10*
Branchio-oto-renal	VUR, renal agenesis, hypoplasia/dysplasia, duplicity, obstruction, HN	Branchial remnant, preauricular pit/tag, microtia, deafness	AD	*EYA1* or *SIX1*
Cat eye	VUR, renal agenesis, hypo/dysplasia, duplicity, HN	Atresia of colon, anus, genitalia, vertebral defects, transesophageal fistula	C	22 partial tetrasomy; inv dup (22) (q11)
DiGeorge/velocardiofacial	VUR, renal hypoplasia, duplicity, HN	Conotruncal CHD, thymic aplasia, typical face, cleft palate	C	*22q11 deletion*
Ectrodactyly-ectodermal dysplasia-clefting	VUR, renal agenesis, renal dysplasia, cysts, HN	Ectrodactyly, hypohidrosis, sparse hair, cleft lip/palate	AD	
Epstein	VUR	Thrombocytopathia, nerve deafness, cataract	AD	*MYH9*
Goldenhar (oculo-auriculo-vertebral)	VUR, renal agenesis, renal dysplasia, HN, duplicity	Hemifacial microsomia, ear anomalies, vertebral defects	S, AD	
Hypoparathyroidism-deafness-renal dysplasia	VUR, renal hypoplasia, renal aplasia	Hypoparathyroidism, deafness	AD	*GATA3*
Kabuki	VUR, horseshoe kidney, duplicity, HN,	MR, Kabuki-like face, large ears, cleft palate	U	
Kallmann	VUR, duplicity, renal agenesis	Anosmia, cleft lip/palate, hypogonadotrophic hypogonadism	XL, AD, AR	*ANOSMIN-1*
Nager acrofacial dysostosis	VUR, renal agenesis, HN, duplicity	Facial bone hypoplasia, cleft eyelid, radial ray defect	AD	*ZFP37*
Polydactyly-obstructive uropathy	VUR, HN, ureteral/urethra diverticulae	Postaxial polydactyly of hands and feet	U	
Renal-coloboma	VUR, renal hypoplasia/dysplasia, renal agenesis	Optic nerve coloboma, nerve deafness	AD	*PAX2*
Urogenital adysplasia	VUR, renal agenesis, renal hypo/dysplasia, HN	Abnormal uterus, deformity of feet and hands	AD	
Renal/Müllerian hypoplasia	VUR, horseshoes kidney, renal hypoplasia	Absent uterus, broad forehead, DD, large fontanel	AR	
Townes-Brocks	VUR, renal agenesis, renal dysplasia, duplicity, ureteral/urethra diverticulae	Triphalangeal thumb, imperforate anus, skin tag, deafness	AD	*SALL1*
Wolfram	VUR, HN	Diabetes mellitus/insipidus, optic atrophy, nerve deafness	M	*WFS1-2*

*AD* autosomal dominant, *AR* autosomal recessive, *C* chromosomal, *CHD* congenital heart disease, *DD* development delay, *HN* hydroureteronephrosis, *M* mitochondrial, *MR* mental retardation, *S* sporadic, *SS* short stature, *U* uncertain, *XL* X-linked (modified from [2]).

## Mendelian inherited syndromes with renal hypo/dysplasia and VUR

Renal-coloboma syndrome (RCS; OMIM 120330) is an autosomal dominant disorder characterized by optic nerve coloboma and renal anomalies, which commonly include renal hypoplasia and VUR. Auditory, CNS, and skin/joint anomalies may also be present. Renal and/or ocular manifestations are found in all patients, although a marked phenotypic variability has been observed. Sanyanusin first described heterozygous mutations of the *PAX2* gene in patients with renal-coloboma syndrome [12]. An experimental model revealed that *Pax-2*−/− mutant mice lack kidneys and a ureter because the ureteric bud fails to branch from the mesonephric duct, while haplo-insufficiency (a lack of functional protein due to a mutated allele) in *Pax-2*+/− mutant mice is associated with renal hypoplasia and sometimes hydro-ureteronephrosis consistent with the presence of vesico-ureteric reflux [13]. Human *PAX2* gene maps to chromosome 10q24–25 and comprises 12 exons. The first four exons encode a highly conserved DNA-binding domain, essential for transcription factor function, whose role during kidney and ureter development has been discussed above. To date, many different *PAX2* mutations have been reported in humans, most frequently affecting exon 2 (https://doi.org/www.hgu.mrc.ac.uk/Softdata/PAX2). Phenotypic variability has been observed not only with differing mutations, but also with the same mutation and within the same family, and *PAX2* mutations have also been reported in isolated renal hypoplasia and in VUR [14].

Branchio-oto-renal syndrome (BOR; OMIM 113650) segregates as an autosomal dominant disease with incomplete penetrance and variable expressivity. Common phenotypic features comprise deafness, preauricular pits/tags, branchial and external ear malformations, and renal anomalies including renal hypo/dysplasia with abnormalities of the collecting system and VUR. In 1997, Abdelhak identified mutations in a novel gene called *EYA1* in seven affected patients [15]. In further reports, 51 different mutations of *EYA1* (https://doi.org/www.medicine.uiowa.edu/pendredandbor) have been associated with BOR syndrome, including point mutations and complex rearrangements, which may involve every exon and seem to be randomly scattered throughout the gene. There is no single common mutation, but most mutations are unique to individual families. *EYA1* maps on chromosome 8q13.3 and is the human homolog of the Drosophila “eyes absent” gene, which is involved in the development of all components of the inner ear. In the developing kidney, the gene is required for metanephric development, as reported above.

*EYA1* mutations are detected in approximately 40% of patients with clinical diagnosis of BOR syndrome. An additional gene, *SIX1* (*Drosophila sine oculis* homologue), which interacts with *EYA1* and *PAX2* in a complex network of genes that control the development of kidneys, ears, and other organs, has been identified [16].

Townes-Brocks syndrome (TBS; OMIM 107480) is an autosomal dominantly inherited disorder, which comprises multiple defects, including imperforate anus, preaxial polydactyly, and/or triphalangeal thumbs and dysplastic ears. Further manifestations include renal anomalies such as hypo/dysplastic kidney and urinary tract malformations, foot malformations, congenital heart defects, and, rarely, mental retardation. Among urinary tract malformations reported in TBS, VUR has also been described [17]. Significant intra- and inter-familiar phenotypic variability has been noted and clinical features overlapping with BOR syndrome have been reported. TBS has been shown to result from mutation in the *SALL1* (*Drosophila sall* homologue) gene, which maps on chromosome 16q12.1 and encodes a zinc finger protein thought to act as a transcriptional repressor (see above). The gene comprises three exons, containing four highly conserved double zinc finger domains. To date, 35 different mutations have been reported, mostly frameshift mutations occurring in exon 2 and found 5′ to or within the region encoding the first double zinc finger. *SALL1* mutation analysis in TBS has failed as yet to demonstrate a clear genotype–phenotype correlation. One hypothesis is that *SALL1* mutations cause TBS via haplo-insufficiency, as most of the observed mutations insert premature termination codons [18]. However, *Sall1* heterozygous knock-out mice showed normal phenotype, while homozygous mutants had isolated kidney defects without other TBS manifestations. In other animal models, heterozygotes already showed a TBS-like phenotype and TBS was considered to be caused by mutations leading to truncated protein with a dominant negative effect.

In hypoparathyroidism-sensorineural deafness and renal disease syndrome (HDR; OMIM 146255), urinary manifestations include renal aplasia or hypoplasia, pelvicaliceal deformity, and VUR. The type and severity of clinical manifestations may range over a wide phenotypic spectrum. The syndrome has an autosomal dominant pattern of inheritance, even though an autosomal recessive mode of inheritance has also been hypothesized. Hasegawa first described HDR syndrome in a girl with a de novo 10p deletion [19]. Subsequent molecular deletion analysis defined a critical region for the disease on 10p14–15. In this context, a zinc finger transcription factor, GATA3, has been mapped. Human GATA3 expression has been detected in the developing parathyroid glands, inner ears, kidney, thymus, and CNS. Gata3 null mutant mice show renal hypoplasia and developmental defects in structures derived from cephalic neural crest cells. Heterozygous human GATA3 abnormalities predicting a loss of function, namely one nonsense mutation, two intragenic deletions, and two whole gene deletions, have been identified in several families with HDR syndrome, suggesting that the haplo-insufficiency of GATA3 is the major cause of the disease [20].

### Primary isolated VUR: candidate gene identification by family studies

The increased sibling recurrence rates as well as parent–offspring transmission strongly implicate hereditary factors in the development of primary isolated VUR. The severity of VUR varies between affected individuals in a family and abnormalities often improve or can entirely resolve with age, making complete ascertainment of affected family members difficult. Moreover, asymptomatic individuals are rarely screened, as the diagnosis often requires invasive procedures. Finally, establishing the diagnosis of reflux nephropathy is equally problematic.

Nonetheless, familial clustering of isolated primary VUR and/or reflux nephropathy have been described and a broad range of inheritance patterns have been reported from family studies, including autosomal-dominant with incomplete penetrance, autosomal-recessive, sex-linked, and even multifactorial. Only Chapman et al.’s study used complex segregation analysis to determine the most likely genetic model. In that set of families, a single dominant gene was best able to explain the transmission of the trait [21].

More recently, multigenerational families with both VUR and reflux nephropathy were examined by genome-wide linkage analysis by Feather et al. In 5 of the 7 families analyzed, VUR mapped to a locus on chromosome 1p13 [22]. In the other two families, no linkage was shown to this region, implicating additional genomic loci in VUR, and 12 additional loci were identified genome-wide with *p* > 0.05. No significant linkage was found to 6p, an HLA locus hypothesized to increase the susceptibility to infection and progression to reflux nephropathy, or to *PAX2*.

A candidate approach to test the association of primary VUR with already reported loci (1p13, 3p12, 6p21, 10q26, and 19q13) did not yield any significant LOD scores [23].

Other authors identified a 13q deletion in several children affected by VUR and reported congenital anomalies of kidney and urinary tract (CAKUT)-associated locus on 13q12–22. These authors very recently described the results of cyto- and molecular genetic studies to identify a second region on chromosome 13 that is located on 13q33–34 and is associated with CAKUT such as severe VUR and reflux nephropathy [24]. The critical region spans approximately 7 Mb and includes 33 genes; in murine models, several of these genes show mRNA expression patterns that suggest their potential involvement in renal/urinary tract development anomalies.

Literature data support the hypothesis that VUR is genetically heterogeneous and is caused by different genes acting either alone or in combination, along with random environmental effects.

### Is VUR a multifactorial complex disorder?

Multifactorial disorders result from complex interactions between a number of predisposing factors including the genotype and a variety of environmental exposures that trigger, accelerate, or exacerbate the disease process. In contrast to mendelian single-gene defects, there is only a handful of multifactorial diseases whose underlying genetic model is known. The major approach used to locate and identify genes that predispose to complex disease is association study, which compares the frequencies of particular alleles between affected and unaffected individuals, enabling the identification of high- and low-risk persons with a predisposition to complex disorders. This genetic analysis is performed using different polymorphisms (DNA variations) that are present at least in 1–2% of normal individuals. In humans, association studies in the VUR-affected population have been performed by polymorphisms of the genes involved in kidney and urinary tract development previously identified in animal models [25].

The renin angiotensin system (RAS) appears to play an important role in renal development [26]. Angiotensinogen mutant mice have atrophic papillae with pelvic space enlargement, chronic interstitial inflammation and fibrosis, as well as ACE (angiotensin converting enzyme) and angiotensin II type 1 receptor (*AGTR1*) null mutant mice. Conversely, angiotensin II type 2 receptor (*AGTR2*) null mutant mice have urological disorders, including both obstructive anomalies and reflux, with or without renal hypo/dysplasia. On the basis of these experimental studies, several authors have hypothesized that genetic polymorphisms of the RAS components may be important factors in VUR-including CAKUT development and progression. However, the results of these studies are conflicting: polymorphisms in ACE and AGTR2 have been inconsistently associated with VUR, reflux nephropathy or combined kidney and urinary tract anomalies.

An analogous result has been demonstrated for Uroplakin genes. Uroplakins (UP) are a group of four membrane proteins that are synthesized by mammalian urothelium, the lining of most of the lower urinary tract, including the proximal urethra, bladder, ureter, and renal pelvis. Genetic ablation of *UPIII* causes VUR in mice [27]. In humans, UPIIIa heterozygous mutations cause renal adysplasia and severe VUR was described in two unrelated kindreds, one isolated and the other associated with a persistent cloaca [28]. In a population of 76 VUR patients, 18 single nucleotide polymorphisms (SNPs), seven of them missense, were found in all four UP genes, but only a weak association between the two UP polymorphisms and VUR was observed [29]. This suggests that missense changes of UP genes cannot play a dominant role in causing VUR in humans, although they may be weak risk factors contributing to complex polygenic disease.

## Conclusions

Animal and human studies suggest that VUR and the associated renal dysplasia may result from a dysregulation of the complex gene network that regulates the normal developmental program of the kidneys and urinary tract. Molecular and genetic studies have greatly increased our understanding of VUR, but the human gene(s) responsible for primary VUR remain to be found. Future approaches to gene mapping technology will allow a better understanding of the genetic factors that underlie VUR.

*Study Questions* (Answers appear following the reference list)

Directions: Each of the numbered items is followed by several possible answers. Select the ONE lettered answer that is BEST in each case.

1. What percentage of chronic renal failure in pediatric patients is due to VUR?
  - 10%
  - 40%
  - 25%
2. In which structures are *Gdnf* and *Ret* expressed during kidney development?
  - Both in metanephric mesenchyme
  - In metanephric mesenchyme and in the ureteric bud respectively
  - In the ureteric bud and in the cloaca
3. What is the prevalence of VUR in the siblings of VUR-affected children?
  - 10%
  - 40%
  - 80%
4. Which gene’s mutation is responsible for BOR syndrome in humans?
  - *EYA1*
  - *PAX2*
  - *WT1*
5. What is the prevalence of gene polymorphisms in the general population?
  - <1%
  - >1%
  - >5%

**Answers:**

1. c)

2. b)

3. b)

4. a)

5. b)
